# Determinants of Active and Healthy Ageing in Sub-Saharan Africa: Evidence from Cameroon

**DOI:** 10.3390/ijerph17093038

**Published:** 2020-04-27

**Authors:** Fomba Louisette Naah, Aloysius Mom Njong, Jude Ndzifon Kimengsi

**Affiliations:** 1 Faculty of Business and Management Sciences, Department of Health Economics, Policy and Management, Catholic University of Cameroon (CATUC), P. O. Box 782 Bamenda, Cameroon; lsama55@yahoo.com; 2 Faculty of Economics and Management Sciences, The University of Bamenda, P. O. Box 39, Bambili, Cameroon; mom_aloys@yahoo.fr; 3 Faculty of Environmental Science, Technische Universität Dresden, 01737 Tharandt, Germany

**Keywords:** active ageing, healthy ageing, determinants, employment, community support, housing

## Abstract

This paper examines the determinants and policy implications of active and healthy ageing in Sub-Saharan Africa, taking the case of Bamenda, in Cameroon. Specifically, the study sought to identify and explore the determinants of active and healthy ageing using a mixed-methods approach involving qualitative and quantitative data collection and analysis. Focus group discussions were conducted complemented by a survey (random and snowball sampling) using a structured questionnaire. Narratives and thematic analysis were used to analyze the data generated from the focus group discussion and Tobit regression was employed to analyze the multiple determinants of active ageing by dimensions and on a global scale in Cameroon. Results identified three key dimensions of active and healthy ageing: employment/livelihood options (EL), community support and health (CH) and housing and living in Bamenda (HL). The regression results reveal gender bias in active ageing, a non-effect of education and health on active ageing, and a positive effect of income on active and healthy ageing. This study contributes, among others, to the competence–environmental press theory on active ageing with regards to unbundling context specific determinants of active and healthy ageing. It equally derives policy considerations with regards to gender mainstreaming and the identification of age friendly income earning options to enhance the active and healthy ageing process.

## 1. Introduction

### 1.1. Ageing Concepts

As populations age, there is an increasing need to ensure that this segment of the population stays active and healthy. The World Health Organization (WHO) defines ageing as a gradual and irreversible process that involves changes in tissues and body functions over time. The ageing population will increasingly need special attention, in order to reach not only an increase in life expectancy, but above all, so that they can live with more quality, in line with the tenets of active ageing [1]. The concept of “active ageing”, which requires careful and focused emphasis on cities, has been related to terms such as “healthy ageing”, “successful ageing”, “productive ageing”, “ageing well”, “living well”, “senior wellness”, “compression of morbidity”. These terms all subscribe to the radically non-traditional paradigm of human ageing, which includes gains as well as losses, and which posits possible improvement in future human health despite increasing longevity. These concepts also strive to compress morbidity into a shorter period later in life, and decrease cumulative lifetime morbidity [2,3]. 

Healthy aging has been described as a lifelong process, optimizing opportunities for improving and preserving health, physical, social, mental wellness, independence, quality of life, and successful life-course transitions. It is emerging as a vital key concept, which according to the (4) Active Ageing Policy Framework implies a focus on the maintenance of health, often through lifestyle choices and preventive measures. Active ageing follows closely from the concept of healthy ageing because only healthy persons both old and young can be active. To promote active ageing, health systems need to take a life course perspective that focuses on health promotion, disease prevention and equitable access to quality primary health care and long-term care. Active ageing is the process of optimizing opportunities for health, participation and security in order for people to attain a good quality of life as they age [4]. The ageing phenomenon is even more visible in growing cities where a large number of the human population is found [5]. It therefore holds that addressing the issue of active and healthy ageing requires careful and focused emphasis on cities. WHO, in its bid to engage and assist cities to become more “age friendly”, prepared the global age friendly cities checklist which consisted of eight domains of urban life: outdoor spaces and buildings, transportation, housing, social participation, respect and social inclusion, civic participation and employment, communication and information, community support and health services [5]. 

### 1.2. Determinants of Active Ageing

**Figure 1:**
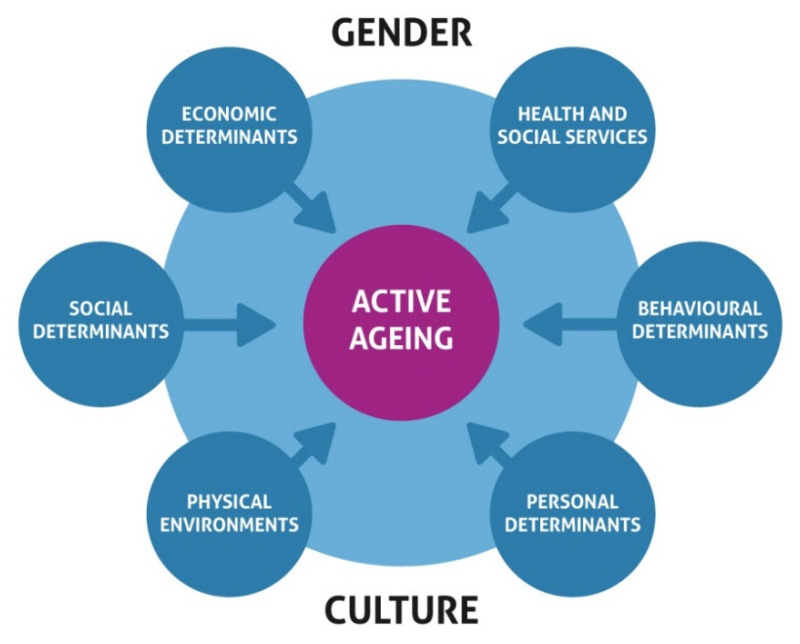
Determinants of active ageing. Source: [7].

The concept of age-friendly cities (communities), represents a place that encourages active aging by optimizing opportunities for health, participation and security in order to enhance quality of life as people age. It is a place that works to improve the livelihood of people of all ages [6]. Active ageing is a multi-dimensional discipline, with many determinants (Figure 1) and which requires that careful attention be paid to each determinant given their vital contributions to the ageing process.

Determinants related to health and social services promote health systems that focus on health promotion, disease prevention and equitable access to quality primary health care and long-term care. Three aspects of the economic determinants are income, work and social protection. Determinants related to the social environment involve social support, opportunities for education and lifelong learning, peace, and protection from violence and abuse. Physical determinants examine the physical environment, personal determinants focus on individual genetics, while behavioral determinants address healthy lifestyles and active participation in one’s own care. Culture, which surrounds all individuals and populations, shapes the way in which we age, gender is a “lens” through which to consider the appropriateness of various policy options and how they will affect the well-being of both men and women [2]. Campos et al. [8] constructed an indicator of active aging and assessed its association with quality of life and possible determinants according to gender. They employed the Aging; Gender and Quality of Life (AGEQOL) study to interview 2052 individuals aged 60 years and older residing in Sete Lagoas in the State of Minas Gerais, Brazil. Using the multiple logistic regressions at 5% level of significance, they analyzed the association between active aging, quality of life and possible determinants for each gender. Their results showed that most men were in the active aging group (58%), and 51.8% of women were in the normal aging group (*p* < 0.001). The final model shows an association between quality of life (physical and psychological) and active ageing outcomes for both genders. Equally, behavioral and community participation factors were positive predictors of active aging among men, while higher income earning women had a better chance of belonging to the active aging group. Their conclusions point to the fact that quality of life and participation in groups are the main determinants of active aging, while other gender-differentiated factors associated with active aging also exist. Through a systematic review of 83 quantitative and qualitative studies, Annear et al. [9] explored the evidence for environmental influences on older adult health and activity participation, and identified knowledge gaps within literature. Applying a Cochrane-type review procedure, they identified support for both personal and environmental influences on health and activity participation in later life. The reported personal influences include ethnicity and cultural norms, energy and motivation, sex, age, education, genetic heritage, self-efficacy and personal financial circumstances, while the environmental influences on activity participation include climate, level of pollution, street lighting, traffic conditions, accessibility and appropriateness of services and facilities, socio-economic conditions, aesthetics, pedestrian infrastructure, community life, exposure to antisocial behavior, social network participation, environmental degradation, level of urbanism, exposure to natural settings, familiarity with local environment and others.

Even though a variety of factors determine active ageing, many studies on active and healthy ageing have focused on illnesses such as HIV/AIDS, diabetes and high blood pressure [10,11,12], losing sight of the need to effectively incorporate health unrelated determinants to provide a holistic picture of the ageing process and its challenges. Healthy aging should be a useful model that tries to fix traits associated with exceptional longevity, such as genotype [13], draws on empirical and applied clinical underpinnings in diverse areas, such as management of chronic diseases, [14], and a focus on lifestyle choices, social, economic, behavioral and environmental factors that affect the health of a person. Descriptive and evaluative research of older people based on biomedical models neglect the vast heterogeneity in health status, and cannot adequately address the concept of healthy aging [15].

Sadana [16] argues that while social and scientific discourse on healthy ageing and on health equity are increasingly available, there is, from a global perspective, limited conceptual and analytical work connecting both. Through an extensive literature review on the overlap between both topics, and based on data from 194 countries, the authors highlight differences in older adults’ health considering three issues: (i) multilevel factors that contribute to differences in healthy ageing, across contexts, (ii) policies or potential entry points for action that could serve to reduce unfair differences or health inequities and (iii) new research areas to address the cause of persistent inequities and gaps in evidence on what can be done to increase healthy ageing and health equity.

Principi [17] explores the conditions under which older workers who are soon to retire have devised plans that are in line with the active ageing agenda. The authors conducted a sample of 133 older workers who planned to retire within the next 10–12 months in Italy, England, and the United States of America (USA), and employed Active Ageing Index dimensions to gauge the orientation of older people towards their retirement. Their study reveals that the retirement plans of interviewees were substantially consistent with the active ageing perspective. However, some challenges were highlighted, including the need for governments to do more to promote genuine freedom of choice in relation to leaving the labor market, and to provide greater support for informal family careers. Their findings equally highlighted the need to measure active ageing in connection with individual wellbeing, e.g., by including indicators of leisure activities and by considering the re-weighting of employment and informal care dimensions. While their study focused on workers who were engaged in different sectors, our focus will be on those who were employed by organizations, including those who were self-employed. 

A number of scientific studies have demonstrated that the lifestyles of older people, including physical activity, are important not only for health and longevity but also functional ability, which is understood as independence in performing everyday activities [18]. Positive correlations between physical activity and functional ability have observed in studies using cross-sectional data [19,20] and panel data [21,22]. To corroborate this perspective, [23] investigated whether higher physical activity of older people dampens increases in public expenditure, particularly the costs of long-term care. The authors employed the Survey of Health, Ageing and Retirement in Europe (SHARE) database to calculate disability rates based on physical activity performed. Their results suggest that disability rates are significantly lower for older people who are physically active, while the promotion of physical activity may significantly reduce future budgetary burden connected with population aging. Some studies have assessed the 2002 WHO Active Ageing Framework and its application to developing countries. Mapoma [24] employed this approach, using data from Zambia. The author explored the applicable determinants of the active ageing from the WHO framework, and analyzed the influence of HIV/AIDS on active ageing in general. A non-experimental exploratory research design was used to collect data for this paper; 690 respondents (284 males and 406 females) were purposively sampled. He identified economic (income accessibility), health (functional limitations), personal/behavioral (low self-esteem and loneliness) and social (low family and peer interactions) forces as determining agents for active ageing in Zambia. The study, however, suggests the need to further ground-truth, taking into consideration empirical verifications from other contexts. 

De São José et al. [25] argue that despite the ubiquity associated with the concept of active ageing, including the contributions of the active ageing index in sensitizing people and policy makers on the multidimensional and complex nature of ageing well, this tool remains under scrutiny. Anchored on the Theory of Model Ageing and the capability approach, the authors attempt a critical analysis of the active ageing index showing that it was developed with the paradoxical aim of deriving the solution from the problem. It remains under-theorized and still begs for conceptual and empirical clarifications. The index focuses on the measurement of achievements and not capabilities, rendering it an incomplete tool to guide policy making. 

Up to date, there is little or no evidence of studies conducted to answer questions related to the application of the active ageing frameworks in developing countries, including sub-Saharan Africa [24], where most of the Millennium Development Goals (MDGs) targets were unmet [26]. Knowledge on the determinants and policy implications of healthy and active ageing still begs for conceptual, methodological and policy edification. Such an unwanted situation accounts for poorly conceived and/or weak program interventions geared towards promoting healthy living among the old. 

Most research on ageing in Cameroon is mostly biological and is focused on curing age-related disease or preventing the incidence of a particular condition in specific risk groups rather than focusing on issues of health in a more holistic manner. For instance, studies on ageing have focused on illnesses such as HIV/AIDS, diabetes, high blood pressure and more [10,11,12]. These studies have all lost sight of the need to effectively incorporate health unrelated determinants to provide a holistic picture of the ageing process and its challenges. However, there has been increasingly overwhelming evidence suggesting the need to budget with health unrelated factors such as lifestyles, built environment and social inclusion [27,28], which, in turn, influences the health and wellbeing of older people.

The case of Cameroon is pertinent. Cameroon is one of the countries that is yet to enact a policy on ageing. Research on this subject is required to provide a comprehensive knowledge base that can contribute to inform its policies. This study aims to bridge knowledge and policy gaps in this regard by analyzing the multiple determinants of active and healthy ageing in the rapidly growing city of Bamenda, Cameroon. Specifically, this study seeks to i) investigate the determinants of active and healthy ageing, and ii) highlight policy implications linked to the active and healthy ageing process in Cameroon. 

## 2. Materials and Methods

### 2.1. Study Area

**Figure 2:**
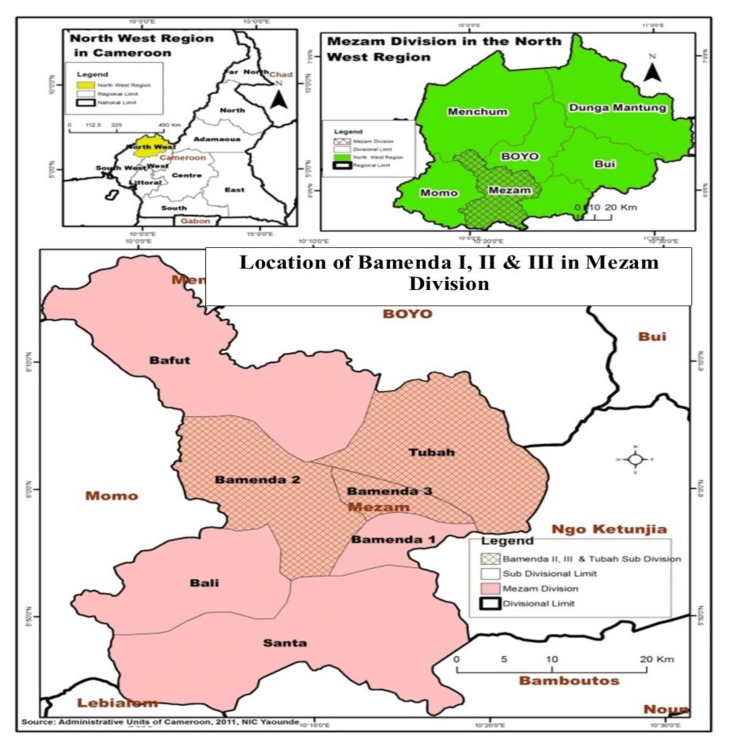
Map of Bamenda in the North-West Region of Cameroon. source [29].

This study is carried out with a group of older persons (men and women 60 years and over) in the city of Bamenda, Cameroon (Figure 2). Cameroon shares land borders with 6 countries: Congo, Chad, Central African Republic, Equatorial Guinea, Gabon, Nigeria (https://www.worldatlas.com/af/cm/where-is-cameroon.html) Bamenda, is located in North-Western Cameroon and is the capital of the North-West Region. The city has an estimated 900,000 inhabitants and is situated 366 km north-west of the Cameroonian capital, Yaounde. Bamenda was purposely chosen in the context of Cameroon, selected based on the fact that it ranks among the major cities in the country. This study is carried out within the three councils: Bamenda I, Bamenda II and Bamenda III councils from which elderly persons were randomly selected. The three council areas in Bamenda are made up of seven villages, Bamenda I (Mendakwe), Bamenda II (Mankon, Chomba, Nsongwa, Mbatu), and Bamenda III (Nkwen, Bandjah). Out of these seven villages, three were randomly selected for the study. The random draw led to the selection of Mendakwe, Mankon and Nkwen. The total population of the aged in the three selected study sites stands at 4000. From the 4000 population, a 10% sample size was drawn constituting 400 respondents for the survey. 

### 2.2. Methodology 

Six focus group discussions were conducted within the 3 study sites. For the focus group discussion, a multi stage sampling technique was used in selecting the sample. In the first step, Bamenda was purposively chosen in the context of Cameroon, selected based on the fact that it ranks among the major cities in the country. In the second step focus group participants (elderly persons) were randomly identified, while the snowball approach assisted in the further identification of other participants to join each focus group. A focus group discussion guide was developed and pilot tested with twenty elderly represented households in Bamenda. The feedback from the pilot test was used to further revise the guide. The focus group discussion guide consisting of three parts was administered as follows: the first part of about 25 min was meant to familiarize participants with the relevant concepts of active and healthy ageing, and to get them more engaged in the discussions. This facilitated a free and open discussion, getting participants to think about their own experience. Key questions centered around the meaning of “good quality of life”, being an older person in a community, and their perceptions of an age friendly community. After a break, the second part of the discussion of about 40 min then visited the 8 domains of the Vancouver protocol age-friendly city methodology, asking participants on each domain about the main barriers and opportunities they observe and the recommendations for improvements that they would make. In this section, we also sought to know if there was any other domain that they would have liked to add to the existing eight domains. The last section, of about 25 min, examined gender differences in the perception and experiences of older men and women, the vision of an ideal community in which to grow old and priorities for action. It was deemed necessary to bring in gender issues into the discussions since existing literatures suggest that active ageing is gender sensitive.

Six focus groups with elderly persons 60 years and above were conducted within one month. Since Bamenda is made up of three council areas, each council area had two focus group discussions (a male group and a female group). All focus groups were facilitated by the researcher and a note taker. Each focus group discussion was audio recorded with the informed consent of participants. Focus group participants spoke in English and in Pidgin English. At the end, we listened to the audio to update or correct notes taken. The information was then presented and discussed under the eight domains. Three out of the eight WHO age friendly city domains (healthcare, housing and employment), were highlighted by seniors in Bamenda as priority areas for them. The feedback from the focus group discussions helped in the selection of the sampling of the sampling approach to employ, and in refining the structured questionnaire. An amount of 400 elderly persons were sampled within the selected villages in the three sub units of Bamenda for the survey, using both random and snowball sampling techniques. 

This survey was an interviewer-administered survey because most of the respondents were not well educated, and although they could speak English, their skills of reading and writing were not very good, and some people could not see well and some were just tired. Surveys were administered through the community health center, households, local businesses, churches and on the streets. This survey method was effective in giving a response rate of 100%. The high response rate achieved by administering the survey face to face allows for more reasonable generalizations about the determinants of healthy and active ageing in Cameroon. Reliability was ensured through pre-testing of the questionnaires to ensure clarity of the test-items through the focus group discussions. In addition, reliability of test items was tested by means of Cronbach’s coefficient alpha (*α*), expressed as [30]:

(1) $$\alpha =\frac{kr}{1+\left(k-1\right)r}$$

This is based on the number of items on the survey (*k*) and the ratio of the average inter-item covariance to the average item variance (*r*), [see Cooper and Schindler 2006]. The Cronbach’s alpha values range from 0 to 1. Estimates of alpha can take any value less than or equal to 1, including negative values, although only positive values make sense. To interpret the Cronbach’s alpha the following rule of thumb are used (Table 1).

### 2.3. Ethical Approval

A research authorization was issued by the Graduate Research Coordinator at the Catholic University of Cameroon (CATUC), and this was then used to request for further administrative clearance with the sub divisional officer for Mezam. In addition, the Regional Delegation of Public Health was contacted to issue health clearance in this regard. The researcher explained the reasons for the study, and sought the consent of the respondents to participate in data provision. Additionally, the consent of the respondents was sought before their views were audio recorded. All respondents willingly agreed to participate and they were assured of the confidentiality of all information that they would give as well as their identities. This assurance made all those who were contacted for both the survey and focus group discussions to provide the information without fear of being exposed. These ethical values applied by the researcher are in keeping with the principles of free prior informed consent, and anonymity.

### 2.4. Methods of Data Analyses

The first phase of data analysis was qualitative analysis involving narratives and thematic analysis with an emphasis on dimensions of active and healthy ageing in line with the WHO framework. The initial coding of information collected during the focus group discussions was completed by the researcher and the note taker. This was checked by the researcher and the second author. Thematic analysis provides an easily accessible and theoretically flexible approach in qualitative data analysis [32]. In this case, the eight domains listed in the WHO age friendly cities guide were discussed with the elderly persons and based on the recurrent themes, they were presented in a tabular form, with a focus on the challenges linked to the eight domains, and the recommendations proposed by participants. This then gave room for further itemization of the three domains which were of critical importance to the elderly persons in Bamenda. Discussions however, were not limited to the eight domains as participants were asked to identify other issues that were not covered by the eight domains. Participants strongly advocated for the support of other livelihood options in a situation where formal employment, was not possible. Livelihood in this case revolved around farming, petit trading and other off-farm activities. Livelihood questions were incorporated within the questionnaire for the survey to cover this aspect captured during the focus group discussions. Another topic discussed out of the eight domains was gender differences in the perception and experience of older men and women. The key issue raised here was elderly men socialize more and are financially independent than women. Hence, they enjoy leisure time more in old age than women. This further informed the empirical analysis on whether active ageing was gender biased.

In the second phase, data collected by questionnaire were sorted manually according to the sub units. The questionnaire was then given codes and the meaning of each code into the statistical package for social sciences (SPSS) software. After the data coding was completed, each questionnaire was then entered into the SPSS data view spreadsheet, completing the questionnaires for each sub unit. The collected data were analyzed using the SPSS software version 20.0 (IBM Corp. © 2011, Armonk, NY, USA,) as well as Microsoft excel 2010 (Microsoft Corporation © 2011, Washington, DC, USA,). We built active aging indices by dimension and globally using the Multiple Correspondence Analysis (MCA) approach. The third phase was the econometric analysis where determinant of active and healthy ageing with a focus on age, gender, education, health and income. The variables and measures of active ageing are presented in Table 2. 

#### 2.4.1. Multiple Correspondence Analyses (MCA)

Since active aging is conceptualized as a multidimensional construct, it is measured through the aggregation of the different deprivation variables experienced by the individuals. Accordingly, capturing active aging involves the aggregation of information provided by several variables into a composite active aging index. The ultimate aim of MCA is to generate a composite indicator of active aging for each individual. A composite index constructed using MCA has a tendency of being negative in its lowest part. This would make interpretation difficult. To bypass this, we normalize the index to make it positive with values ranging from 0 and 1 [33].

#### 2.4.2. Tobit Regression Model Specification

To identify the factors that correlate with active aging, we employed a censored regression analysis—specifically, a Tobit model. Known also as the censored regression model, the Tobit model estimates linear relationships between variables that depict either left- or right-censoring in the dependent variable (equally referred to as censoring from below and above, respectively). Our use of the Tobit regression model is justified on the grounds that the active aging indices (employment, community support and health, and housing and living), which are the dependent variables, range from 0 to 1. The Tobit model is a censored normal regression model, applicable in cases where the dependent variable is constrained in some way. In our case the dependent variable (the active aging index) is observed for values greater than 0 but is not observed (that is censored) for values of 0 or less. Additionally, values greater than 1 cannot be observed. Therefore, our model has limiting values at 0 and 1 and only observations between these limits are considered. The structural equation in the Tobit model is expressed as follows [34].

(2) $$Y_{i}^{*}=\beta_{i}X_{i}+{ℇ}_{i}$$

where β is a vector of unknown coefficients, *X* is a vector of independent (predictor) variables (age, level of education, gender, health status, income), and ℇ is an error term that is assumed to be independently normally distributed. 

Y^*^ is a latent variable that is observable. If data for the dependent variable are above the lower limiting factor, zero in this case, Y^*^ is observed as a continuous variable. If Y^*^ is at the upper limiting factor, it is held at one. This relationship is presented mathematically in the following equation:

(3) $$Y_{i}=\{\begin{array}{c} Y^{*}\;if\;\;Y_{i}>0\;\\ 0\;\;if\;\;Y_{i}\;\leq 0 \end{array}$$

*Y* is the observed dependent variable (e.g., employment, community support and health, and housing and living). The Tobit model is non-linear and its estimation is completed using the maximum likelihood method. The explanatory variables of the regression include the age of the respondent, the level of educational attainment, gender, the current health status of the individual and whether the individual has a regular monthly income or not. The dependent variable is the composite ageing indices whose values range from 0 to 1. Coefficients from Tobit regression analysis are not readily interpretable as effect sizes. Our interpretation of these coefficients focused on the sign of the coefficient and whether it is statistically significant or not. 

## 3. Results

Discussions by 100 participants with demographic data on Table 3, were based on the WHO age friendly cities guide (Table 4). It was concluded that not all eight domains of the guideline were of immediate interest to the seniors in Bamenda, pointing out three priority domains for which action needs to be taken to make a difference in the quality of life of elderly persons in the city of Bamenda. 

Firstly, there was a great desire to improve healthcare for the elderly (eighth domain). Secondly, they identified the promotion of livelihood activities for the elderly (sixth domain) and thirdly, building houses appropriate to older persons who do not own houses and build low cost houses for the elderly was raised (Third domain).

### 3.1. Challenges Linked to the WHO Age Friendly Cities Guide 

From the focus group discussions, eight domains listed in the WHO age friendly cities guide were discussed with the elderly persons in the city of Bamenda. The results from the focus group discussions are presented in Table 5. Challenges that were highlighted under each domain are presented with corresponding suggested recommendations by participants. Participants explained that outdoor spaces and public buildings have a major impact on the mobility, independence and quality of life of older people and affect their ability to “age in place”. They cited the lack of sidewalks on many roads within the city, lack of public restrooms in almost all areas of the city, the absence of elevators in most public buildings, and reserved parking for the disabled and elderly, as their key challenges under this domain (Table 5). A respondent noted that:

*“I think the city of Bamenda is constructed without taking into consideration anything that is specifically friendly to the ageing...the city of Bamenda is constructed for the young.”- Male Bamenda II.*

Transportation includes accessible and affordable public transport, and easy transportation determines social and civic participation. Participants noted that public transport was not affordable, safe or convenient. The traffic congestion and disorder in the town was a disincentive for many older people to drive in the city. Even though there was a huge presence of motor bikes that could go into the quarters and take people to their house, they pose a high risk to seniors. 

*“We fear motor bikes more than the taxis ….for us who drive, I don’t know how sometimes we even manage to go through” Elderly woman Bamenda III*.

Respondents raised issues on different aspects of housing structure, design, location and choice and housing was considered as essential for safety and well-being. A few participants were comfortable with the fact that the houses where they lived at the time of the study had all indoor facilities like toilets and kitchens, and were owned by them. They are not required to pay rent, and the housing quality could be improved by their children who were financially stable. A majority of the respondents were challenged by their housing situation. With challenges and recommendations for improvement presented on Table 5, the lack of affordable housing options, housing constructed when occupant was young and now needing adaptation to meet the needs of the old were key issues. Participants believe age-friendly housing should facilitate “ageing in place”.

*“I build my house in my 40s and put facilities which are now not comfortable for me at my age” man Bamenda III*.

Staying connected with events and people and getting timely, practical information to manage life and meet personal needs is vital for active ageing. The availability of many community radio and television stations with programs in Pidgin English, town criers, and cell phones was highly appreciated as a means of communication. The internet services, though challenging to catch up with, were not available to every senior but were a plus to those who had it and were able to use it. Font sizes on newspapers were also too small to read. 

*“The coming of the internet is difficult for us to catch up with the new technology” Man Bamenda III*.

An age-friendly community provides options for older people to continue to contribute to their communities, through paid employment or voluntary work if they so choose, and to be engaged in the political process. Participants were generally satisfied with the opportunities they had for civic participation, but employment and voluntary service opportunities were virtually non-existent and this was linked to the high rate of youth unemployment. Self-employment in agriculture and creating small scale businesses was seen as the only existing option for seniors in the city of Bamenda. 

Health and support services are vital to maintaining health and independence in the community. Participants noted very difficult access to a range of health, community programs and services. Many of the concerns raised had to do with health care costs that are perceived as too high even as participants insisted on the fact that decent ageing can only be achieved when an elderly person can afford healthcare. There was a general conception that seniors were not given any particular attention because there were no specialized health services for the elderly, no geriatric nurses and doctors with particular focus on seniors. Furthermore, participants noted the lack of health information targeting the specific needs of seniors, the absence of emergency health services for seniors, the absence of persons to take the elderly without care givers to the hospital, the lack of social workers to pay home visits to the elderly, especially those abandoned, and no preference given to the elderly in health structures by younger persons and health personnel.

*“People don’t care about the aged when they visit hospitals. You can even sit there and die without somebody attending to you” man Bamenda III*.

Participants were generally not positive about the respect and social inclusion of seniors in the community. A common view was that the respect of the elderly which in the past was a cultural requirement was drastically being lost especially amongst the younger generation. Respect for seniors was tied to health, social and economic status. Quotes were recorded, such as:

*“There is a wide gap between the young and the elderly…I heard a youth telling an elderly person that respect is in the village but that in town it is money that talks” Man Bamenda II*.

*“Cultural values are diminishing----usually in our days when an old man is coming; children are already arranging where he will sit. But it’s not the case today---they think we are old and gone” Man Bamenda II*.

Social participation and social support are strongly connected to good health and well-being throughout life. Study participants were generally not satisfied about their opportunities to participate in activities and events in the city. Older people, especially elderly women, were seen to be very active only in social events like marriages, funerals and in social and professional groups or organizations in which they are members. They regretted the fact that overall, there were no formal events available in the city organized by city authorities for the elderly or in which the elderly could effectively take part. Quotes were recorded, such as:

*“We associate and participate in social and church meetings where our impact is felt” Woman Bamenda III*.

This study identified three priority domains in which action needs to be taken to make a difference to the quality of life of elderly persons in the city of Bamenda. Firstly, there was need to improve healthcare for the elderly with a focus on reducing cost of consultations and drugs for the elderly. Secondly, they identified the promotion of livelihood activities for the elderly, and thirdly, modifying existing houses appropriate to older persons needs and build low cost houses for the elderly. These priority areas were further examined by means of surveys whose outcomes are presented on Table 6 and Table 7. 

### 3.2. Determinants of Active and Healthy Ageing

#### 3.2.1. Active Ageing Indices

The three priority areas were further analyzed to have a clearer picture of the situation. Table 6 indicates that in the employment opportunities/other livelihood activity dimensions, only 27.53% of seniors are able to age actively, which implies that 72.47% of seniors do not have access to employment or support for other livelihood opportunities at the age of 60 years and older. The 27.53% of seniors who can age actively in the employment/livelihood domain represents the small portion of the retired workers who are able to find new jobs upon retirement (teachers and journalists, as reported in the focus group discussions), and business owners who continue with their businesses after the age of 60. Other retired seniors from other professions, who are still active and willing to continue working, are unable to find employment options. The situation is worst for seniors who never had any formal job, had no education, had no privately owned businesses and mostly depended on manual jobs to earn a living. When they become older, and can no longer support those energy demanding jobs to make ends meet, they age with difficulties since no social security cover exist for them and no support for lighter livelihood activities that suit their current needs exist. 

The health care index (Table 6) indicates that only 47.99% of seniors are able to age actively in the healthcare domain, which implies that 52.01% of seniors are unable to have access to healthcare. 

Only 34.82% of older adults can age actively in the housing domain, meaning 65.18% of seniors say that their current housing needs have not been met. The 65.18% of seniors who find it difficult to age actively in the housing domain are those who do not own houses and depend on rentals, thereby wishing they could find low cost houses that meet their current needs, and those who own houses that no longer meet their current needs are unable to modify them to suit their current needs due to financial hardship. 

The global ageing index for Cameroon was 36.73%, meaning 63.27% of older persons in Cameroon are not able to age actively following their preferred dimensions. 

Respondents had ages ranging from 60 to 98 years with an average age of 68.6 years. The results also indicate that 29% of the respondents had no education, 34% had primary education, 25% had secondary education and only 11% had university education. 

Good health was a major concern to respondents, as it enabled them to keep going. The study results revealed that 26.25% of respondents of the survey had poor health conditions, 49.75% had fair health conditions and only 23.25% of respondents reported that their health was good. 

37.75% of the respondents did not have a monthly income and even the 60.75% who had it said it was grossly insufficient. 

#### 3.2.2. Tobit Regressions on the Determinants of Active and Healthy Ageing

The test of global significance which is based on the likelihood ratio (LR) and the Chi2 statistics allows us to judge whether the model taken as a whole is well specified. The null hypothesis of the LR Chi2 states that all the independent variables are not significant in explaining the dependent variable, while the alternative says the reverse. In our case, concerning the Tobit regression model for employment index, our LR Chi2 statistics are equal to 85.31 with a *p*-value of 0.0000 which is clearly less than 1%, showing a 99% reliability. This result is the same for the community support and health index (CH) with a *p*-value of 0.0000 and the housing and living in the city index (HL) with a *p*-value of 0.0002 which both have a 1% level of significance. The model is also globally significant at 1% with a *p*-value of 0.0000 giving it a 99% level of reliability. This level of reliability enables us to present the results. The Tobit regression uses four independent variables in an attempt to explain the dependent variable under the three dimensions being examined. 

The regression results on Table 7 show that globally, with respect to all three dimensions under review, women are less likely to age actively when compared to their male counterparts. The global active ageing index for women with reference to men being −0.03733. This is linked to poverty and the high rates of dependency. This was addressed by some of the respondents during the focus group discussions as follows:

*“Poverty causes me not to go to hospital even when I am sick…. the demands from the family are too much” woman Bamenda II*.

*“I have grand children to take care of; at times, I live on charity from neighbours. I do not have children who can assist me, my only child died. The government does not know that we exist. I am just waiting for my time to die” Woman Bamenda II*.

These results show that seniors with either primary, secondary or university education are not more likely to age actively across all three domains when compared to those with no education. A global index of −0.0815 for primary education, −0.22235 for secondary education and −0.23812 for university education when compared to no education all shows a negative relationship between education and active ageing in the Cameroonian context, in the case of Bamenda. Even seniors with some level of education complained of not having any gainful employment opportunity. This was raised during focus group discussions as follows: 

*“For employment, there is nothing for old people and volunteering is not realistic because we still need money at our age” Man Bamenda I*.

*“Self-employment is common with the elderly, activities like farming, piggery and petite trading are carried out” Man Bamenda I*.

The same results also show that seniors with fair or health conditions are not more likely to age actively across all three domains when compared to those with poor health. The global index for fair health compared to poor health is −0.07797, while that of good health compared to poor health is 0.12059. However, the general tendency of the respondents with regards to what constitutes a good quality of life centered on having good health. As one of the respondents put it: 

*“Good quality of life to me now is having good health which means you must have had reserves to foot present bills”- Man Bamenda III.*

There is evidence from the regression results that seniors with a monthly income flow are more likely to age actively across all three domains when compared to those with no monthly income flow. The global index for no monthly income when compared to monthly income is −0.03591. There are no subventions to provide health care for the elderly in the Cameroonian context. This affects access to health for this group of people, as recounted by one of the respondents: 

*“At my age the demand for money is still increasing even hospital bills are higher and I do not have the strength or energy to do strenuous work ---I still have many responsibilities and many people are still expecting help from me, all these affect my health” Man Bamenda I*.

## 4. Discussion

The objectives of this study were to: i) investigate the determinants of active and healthy ageing, ii) to highlight policy implications linked to the active and healthy ageing process in Cameroon. Gender and income were seen to have a positive relationship with active ageing, while level of education attained did not have a positive relationship with active ageing. Having access to healthcare did not have a positive relationship with active ageing but was desired by seniors in order to live a pain free life and maintain an independent level of daily functioning.

The findings reveal that active ageing is gender biased in favor of men. The study findings on marked divergences in the experiences of older men and women resonate fully with a broad recognition of the gendered nature of ageing and old age in the African and global gerontological literature [35,36,37,38]. More specifically, the picture of older women’s continuing spousal and grand parental roles, and of their closer ties to and greater concern for their offspring, echoes evidence emerging from qualitative research in other African contexts [35,39,40]. In a similar vein, the notion of disparities in older men and women’s health and physical capacity with women at a clear disadvantage, concurs with a body of robust evidence, showing similar gender inequalities in health status and health-related quality of life in old age in various Sub Saharan African settings [41,42,43,44,45,46,47,48,49]. The common perception, that older women’s poorer health is due, in part, to the physical strain associated with their domestic and care roles, chimes broadly with research evidence from SSA on the negative impacts of older persons’ caregiving roles on their physical and mental well-being, particularly in contexts of HIV/AIDS [50,51,52,53,54]. These results are in line with a study by [55], who analyzed the situation of the elderly in Ghana, and concluded that elderly females are more vulnerable and disadvantaged than their male counterparts due to low educational attainment resulting in low female participation in the formal sector. The findings, however, contrast with the findings of [24], who concludes his study by saying that gender does not influence active ageing in Zambia. His finding defies propositions given in the Active Ageing Framework, where gender is an overarching variable influencing active ageing in general, even though culture in his case influences active ageing (*p* < 0.0001; Beta = 179).

The results of our study suggest that there is no positive relationship between level of education attained and active ageing. These results are in contrast with a study by [56], in an article titled Changes in Social Participation of Older Adult in Beijing, which revealed that the higher the level of education of older adults, the stronger the willingness for social participation. He proceeds by saying that better education, to some degree, leads to older adults having more resources available to share and therefore a greater possibility of social participation. Istance [57] argues that there is a case for thinking about learning for older people as part of active ageing and not as part of an overextended and unmanageable lifelong learning strategy that strives to cover all age groups. Despite this, our study suggests that in our context, there is no positive relationship between ageing and educational attainment. Engelbrecht [58] points out that education and training are gaining importance in meeting people development challenges in an ageing population. 

A key barrier which came up strongly and was related to older people’s essential concerns about maintaining their health in the focus group discussions is their present lack of access to quality health services to address their health needs. This was confirmed after the survey with the community support and health index displaying 47.99%, meaning 52.01% of the respondents could not age actively under the health domain. 

In highlighting the nature and key role of access barriers to essential health services, this study resonates with an increasingly large body of evidence and intensifying debate on health and health care for older people in sub-Saharan Africa [50,59,60]. Respondents expressed concerns on about their inability to maintain their health and functioning. Their concern pertains, in particular, to their financial capacity to afford the necessary health care, given the substantial out-of-pocket expenditure required to access health services. Maintaining health and functioning, especially among older women, is crucial for fulfilling domestic aspects of their care roles, which, due to the many infrastructural and amenity constraints within their communities, require significant physical capacity. 

Older people’s concerns over their health and functioning contrasts starkly with the largely positive emotional tenor of older people’s experiences in western societies [61,62]. Important differences between African and developed world perspectives emerge in relation to the reasons why health and family relationships are considered essential. Whereas in Africa and other low-income contexts, as exemplified in this study, good health and functioning in old age are critical for maintaining livelihoods of self and dependent kin, their relevance in higher income settings relates more to a continued ability to engage in valued social—including leisure—activities [63,64,65]. Good kin relationships, especially to adult children, are perceived as vital in low resource African settings primarily in terms of the material and long-term support they offer in the area of health, consumption needs, etc. In contrast, their importance in high income contexts centers on their role as sources of emotional closeness, trust and companionship [64,65,66]. Older people in the western world view a maintenance of health and functioning as critical, as it implies a continued capacity for earning incomes or involvement in any gainful economic activity. Unfortunately, maintaining health and functioning in our context does not necessarily lead to a continued capacity for earning incomes which the older people very much need to sustain their own well-being and those of dependent younger children and grandchildren, inside and outside of their households.

The findings of this study regarding concerns over material security, prospects of younger generations and their readiness to provide filial support, are very much in line with existing evidence from investigations in other low resource settings in SSA [39,67,68,69,70]. Just as in the study settings, the contexts within which these concerns have been shown to arise are typically characterized by an absence of comprehensive pension and health insurance systems, underemployment among younger generations who are therefore not positioned to provide sufficient material support to older parents or relatives and who, instead, may seek to rely on the older generation for support. 

Older people’s core concern over material security and prospects of receiving support from their children was viewed as an interlinked bedrock of quality of life in older age. In emphasizing the above factors as essential components of quality of life, the study findings strongly echo existing empirical evidence on older adults’ perceptions from anthropological and sociological studies in other SSA settings [66,68,71,72]. Indeed, while, as [63] notes, notions of well-being in old age are anchored in cultural values and modified by context and circumstance, health, material security and family relationships emerge consistently as core components of older people’s perceptions of quality of life across developed and developing societies [61,66,73]. 

The findings of this study relate with [74]’s Theory of Insideness, which conceptualized attachment to place. Older persons in this study increasingly become attached to the place where they live and have a clear preference to continue living where they have been living. The study also relates positively with the Continuity Theory of Ageing as stated in [75], where individuals do not really change as they age, but become more of what they have always been. This was seen especially with women who continued with their care roles in the family as they grew older. The men, however, were restricted from continuing with socialization, largely because of limited financial capacity. According to the Competence–Environmental Press Theory introduced by [76], in order to age-in-place, it is necessary that the immediate, as well as the near environment, is free of barriers that can hinder independent functioning. This mostly applies to western countries, but in our study area, Bamenda in Cameroon, even when barriers are not removed from the environment, older persons still prefer to age in place, and try to deal with the environmental challenges in their own way.

The paper suggests that there is no positive relationship between level of education attained and active ageing. This contributes to another line of thinking; especially as most previous studies argue that there is a positive relationship between active ageing and level of education attained [56,57,58]. In addition, this study showed that having or not having an education in old age in Cameroon does not guarantee more resources in old age due to a new employment or job. The problem of unemployment in Cameroon, especially for the younger generation, keeps the older generation far behind on the queue for employment. Therefore, the level of education attained does not enable employment or more money to spend at old age. We therefore argue that the relationship between the level of education attained and active ageing depends on the context under which the study is carried out and does not necessarily qualify as a general rule. Furthermore, by revealing that older adults in fair or good health are not more likely to age actively when compared to those with poor health, this study contrasts existing literature [61,62,63,64,65]. Regarding the determinants of active and healthy ageing, very few studies in Sub-Saharan Africa, especially Cameroon, have explored these determinants from the lens of gender, income security, educational level attained and health status. Through a mixed-methods research approach, this study contributes in this area, especially as previous studies have mostly used either a quantitative approach or a qualitative one [77,78,79].

## 5. Policy Implications

The United Nations Sustainable Development Goals, specifically Goal 3, calls for the need to “ensure healthy lives and promote wellbeing for all at all ages”. This goal can only be achieved through the setting and attainment of country specific targets. However, policy orientation to fill such gaps requires significant scientific research to unravel the patterns, determinants and challenges involved in designing appropriate policies for active and healthy ageing. This study therefore makes a contribution in this regard by highlighting the fact that, in the case of Cameroon, the health and wellbeing of its seniors greatly depends on the orientation of an ageing policy environment that provides gainful and age-friendly income generating opportunities, since older adults with no income were less likely to age actively when compared with adults with a monthly income flow. 

Given that a significant number of older persons are not employed and, consequently, do not receive retirement benefits, policy focus should consider supporting the aged technically and financially, to engage in income generating activities. This should be championed by state actors, specifically, the Ministry of Small and Medium Sized Enterprises, Social Economy and Handicraft (MINPMEESA), in partnership with the city council. Seniors could also be empowered financially by improving their access to finance through Micro-Finance Institutions (MFIs). Limited access to finance is a common barrier for older persons to start new enterprises due to discrimination which in part is because of their age and in a large part due to their lack of collateral assets. New policies or revision of existing policies should oblige MFIs to put programs into place that favor access to finance for older persons as part of their Corporate Social Responsibility (CSR). This will help older persons to integrate in the labor market through an income-generating activity either in the form of a start-up or through increased employability. Targeted microcredit programs are needed to make microcredit more socially inclusive, as there are signs that microcredits in Cameroon do not adequately serve the needs of the elderly. The provision should be accompanied by proper guidance and follow up to ensure their effective investment in the business which the older person has indicated interest in.

The focus group discussions revealed a strong connection between inadequate income flow and the increasing use of traditional medicine and self-medication which is very detrimental to the health of seniors. Government policies should focus on providing discounts for health bills of elderly persons, creating consultation days or hours dedicated to the elderly, create elderly waiting spaces in the hospitals, train geriatric nurses and doctors and integrate palliative care approaches into older patient care plans alongside active treatment. 

This study concluded that a significant majority of older persons are less likely to age actively in the housing domain. They face challenges of modifying their homes to accommodate their changing needs as they grow older and, in addition, challenges of finding affordable houses that are age-friendly. It is therefore necessary for government and the city council to have policies directed towards increasing the supply of affordable, adaptive and accessible houses for the elderly and to modify existing accommodation for seniors to more age friendly living spaces. Building permits approved by the city council should consider age friendly housing.

Policies to empower seniors should favor women more than men, because this study shows that women are less likely to age actively than their male counterparts. Issues raised from focus group discussions that lead to the disadvantages position of ageing women and which could form a base for policy considerations include restriction of education of the female child, childbirth without adequate healthcare and support, caregiving responsibilities associated with mothering, grand mothering and looking after one’s spouse and older parents that prevent or restrict working for an income and access to an employee based pension, widowhood which commonly leads to a loss of income and may lead to social isolation. Policies directed towards supporting female child education, healthcare and financial support associated with child bearing, financial support associated with care roles and policies that uphold the rights of widows could improve the quality of life of women as they age.

## 6. Conclusions

Based on the findings, the following conclusion can be drawn. Of all the eight dimensions of active and healthy ageing, as outlined by the WHO, three are of great concern to older persons in Bamenda. These include employment, health and housing.

Of these three, the issue of employment was highlighted as the most essential. This is explained by the fact that being employed implies income to sustain an improved quality of life for the elderly. Income can lead to better housing and better healthcare.

Although employment represents a crucial active ageing dimension, a greater majority of the old population do not age actively under the employment dimension. Therefore, targeted interventions are required to secure age friendly employment opportunities for older persons.

A slight majority of the elderly are less likely to age actively in the health domain. This suggests that the health facilities are either inadequate or not easily accessed by the elderly. A strong connection exists between limited access to health facilities and the increasing use of traditional medicine and self-medication.

Housing quality is a function of a number of determinants including income. In this study, we conclude that a significant majority of older persons are less likely to age actively in the housing domain.

Considering gender as a determinant, women are less likely to age actively than their male counterparts. The level of education at old age has no significant effect on active and healthy ageing. There was no significant difference between older persons who were in good health and those who were not considering the employment and housing domain, suggesting that the health status has no effect on active ageing.

Having a monthly income determines active ageing. This led to the conclusion that there was a positive relationship between income and active ageing. Future research should profoundly consider (1) comparatively analyzing socio-economic determinants of active ageing across two or more Cameroonian cities, and (2) investigating differences in active ageing across socio-cultural groups (including vulnerable and minority groups).

## Figures and Tables

**Table 1:** Accepted rule of thumb for interpreting the Cronbach’s alpha.

Crombach’s Alpha	Interpretation
α ≥ 0.9	Excellent
0.9 > α ≥ 0.8	Good
0.8 > ≥ 0.7	Acceptable
0.7 > α ≥ 0.6	Questionable
0.6 > α ≥ 0.5	Poor
0.5 > α	Unacceptable

Source: George and Mallery (2003, p. 231). [31].

**Table 2:** Variables and measures of active ageing indices and socio-economic determinants.

Variable	Indicator	Measurement Unit	Min/Max Score
Employment (Emp)	Gainful employment (employed, unemployed)	1 = employed 0 = otherwise	Min = 0 Max = 1
Community support and health (CH)	Health status (good, poor)	1 = good 0 = otherwise	Min = 0 Max = 1
Housing and Living in the city (HL)	Living space (own house, does not own a house)	1 = own house 0 = otherwise and others	Min = 0 Max = 1

**Table 3:** Characteristics of focus group participants.

Characteristics	N°	Characteristics	N°
Gender		Marital status	
Men	52	With spouse	56
Women	48	Widower /Widow	44
Total	100	Total	100
Age		Health status	
60-69	63	Poor	24
70-79	34	Fair	49
80+	3	Good	27
Total	100	Total	100
Home		Council area	
Own home	73	Bamenda I	31
Rental/Others	27	Bamenda II	34
Total	100	Bamenda III	35
Education		Total	100
No education	24		
Primary	39		
Secondary	27		
University	10		
Total	100		

**Table 4:** World Health Organisation (WHO) age friendly cities guide.

	Age Friendly Criteria	What Each Criterion Covers
1	Outdoor spaces and buildings	Public areas, pavements, outdoor safety, public toilets, green spaces
2	Transportation	Public transport cost and accessibility, taxis, roads
3	Housing	Sufficient and affordable housing, modifications
4	Social participation	Venues, events and activities, information, cost, outreach
5	Respect and social inclusion	Consultation, visible in media, recognized and respected in community
6	Civic participation and employment	Volunteering, paid work opportunities, training
7	Communication and information	Information for all ages, appropriate printed info, phone answering services
8	Community and health services	Adequate health and community services, home care, information

Source: WHO [6].

**Table 5:** Outcome of focus group discussions.

**Outdoor Spaces and Public Buildings**	
Challenges	Recommendations
Lack of rest areas along sidewalks Lack of public restrooms. No streets and house numbers No street lights in the quarters Lack of sidewalks, and inaccessible public buildings No lifts in most public buildings Slippery streets and buildingsNo reserved parking for the disabled and elderly	Build public toilets Provide sitting benches Improved cleanliness of city Repair roads and provide sidewalks Number houses and streets Sensitize architects and engineers on the needs of the elderly City council should give building permits only to age friendly buildings Light the streets in the neighborhoods Increase parks and green spaces
**Transportation**	
Challenges	Recommendations
Narrow and bad roads Bike riders and most taxi drivers do not know the high way code and no refresher courses for drivers. No reserved parking for the disabled and elderly No seats in the few waiting places for caps Overloading taxis and reckless driving. Difficulty climbing on bikes, bikes are risky for the elderly and are involved in many accidents. The disorderly nature of bike riders and the traffic congestion makes it difficult for the elderly to cross the road.	Create bus service for seniors, with specific waiting points and time Provide seats in waiting places for caps Repair roads Traffic police should increase support to the elderly Regulate activities of bike riders in town
**Housing**	
Challenges	Recommendations
Poor drainage system around house High rent payment Slippery bath tubs in houses House very near the road, so lots of noise from cars Rooms for seniors located upstairs in some houses Stairs in the house with no internal toilet facility and small bed rooms	Construct low cost houses for the elderly with indoor facilities, e.g., toilets, not too many windows and no slippery floors. Build a centre to house elderly people who have been abandoned by their family and are homeless.
**Communication and Information.**	
Challenges	Recommendations
Fond sizes in newspapers are very small and not easy to read except with glasses No radio or television programs and information targeting elderly people Internet not accessible to the elderly	Continue giving information to the elderly via the churches Encourage the use of town criers for information dissemination. Produce targeted programs and information for elderly persons by audio visual and print media Increase pidgin English programs
**Civic Participation and Employment**	
Challenges	Recommendations
There is high youth unemployment, so elderly people have limited opportunity to be employed Elderly persons are exploited when employed with pay packages not usually encouraging Rare opportunities for voluntary services and no volunteering spirit in the population Some seniors do not have Identity cards so cannot register to vote Available jobs are strenuous and not adapted to elderly people	Encourage self-employment of seniors in farming and livestock production Encouraged elderly persons to create groups for producing marketable products Create employment for the youths Provide simple employment task for elderly persons which will give them an opportunity to go out and be active Make cash transfers to the elderly to start a small-scale business Encourage income generating activities like small pig or poultry farms or marketing
**Community Support and Health Services**	
Challenges	Recommendations
Healthcare is expensive and seniors lack finances to buy medications Lack of health information Absence of persons to take the elderly without careers to hospital. No home visits to the elderly No specialized health services for the elderly and no geriatric nurses and doctors Absence of emergency health services No social insurance coverage for those who had no formal employment. No preference given to the elderly in health structures	Train health specialist for the elderly (geriatric nurses and doctors) Provide free or subsidized health care to seniors by reducing cost of consultation and medication. Put Policy in place to give preference to elderly people at public places especially in hospitals. Promote “an elderly people adoption scheme”, where well to do persons can support a needy elderly person in the community. Educate population on the needs of elderly people Organize home visits by medical teams to the elderly especially those without caregivers
**Respect and Social Inclusion**	
Challenges	Recommendations
Community respect for seniors is reducing No intergenerational interaction exists except at churches and village gatherings Younger persons are not conscious of the challenges of seniors Wisdom of elderly not exploited by the young Opinions and concern of the elderly is not solicited in public forums Councils have no specific program for the elderly	Organize intergenerational activities where the young will learn from the old how to cook, do needle work, tell stories of the past just to name a few. Provide specific sections for seniors at public places like banks, hospitals just to mention a few. Carry out advocacy for the right of the elderly Educate the young on respect for the elderly in schools and at home for change of mentality and attitude of youths Implement existing laws in the penal code (article 188) that protect the elderly
**Social participation**	
Challenges	Recommendations
Absence of recreational activities provided for seniors by the councils or other organizations Club 58 only for well to do seniors	Create recreational activities for the elderly at age friendly venues and time Create clubs and social centres for the elderly around their neighbourhoods Put in place University of the 3rd age for adult education

**Table 6:** Active ageing indices (descriptive statistics).

Variable	Obs	Mean	Std. Dev.	Min	Max
Dependent variables					
Emp_index	400	0.2753	0.3019	0	1
CH_index	396	0.4799	0.3456	0	1
H_index	397	0.3482	0.2828	0	1
G_index	396	0.3673	0.2306	0.1064	0.8489
Independent variables					
Male	400	0.455	0.4986	0	1
Female	400	0.5425	0.4989	0	1
Age	398	68.6307	6.8893	60	98
age2	398	4757.51	1005.033	3600	9604
No Education	400	0.29	0.4543	0	1
Primary Education	400	0.34	0.4743	0	1
Secondary Education	400	0.2525	0.4349	0	1
University Education	400	0.115	0.3194	0	1
Poor_health	400	0.2625	0.4405	0	1
Fair_health	400	0.4975	0.5006	0	1
Good_health	400	0.2325	0.4229	0	1
Monthly_income Yes	400	0.6075	0.4889	0	1
Monthly_income No	400	0.3775	0.4854	0	1

Where Emp. Index = 27.53%; CH index = 34.82%; HL index = 47.99%.

**Table 7:** Tobit regressions.

	Emp_index	CH_index	H_index	G_index
Male (ref)	-	-	-	-
Female	−0.00366	−0.07308	−0.05790 *	−0.03733 *
	(−0.12)	(−1.47)	(−1.87)	(−1.72)
Age	0.03002	0.00292	0.02463	0.019157
	(1.08)	(0.06)	(0.84)	(0.94)
age2	−0.00018	0.00002	−0.00014	−0.00011
	(−0.93)	(0.06)	(−0.71)	(−0.76)
No education (ref)	-	-	-	-
Primary Education	−0.06584 *	−0.13450 **	−0.10062 **	−0.0815 ***
	(−1.78)	(−2.15)	(−2.59)	(−3.01)
Secondary Education	−0.19308 ***	−0.45943 ***	−0.15179 ***	−0.22235 ***
	(−4.62)	(−6.49)	(−3.47)	(−7.27)
University Education	−0.23210 ***	−0.43565 ***	−0.15624 ***	−0.23812 ***
	(−4.32)	(−4.85)	(−2.79)	(−6.02)
Poor health (ref)	-	-	-	-
Fair health	−0.10758 ***	−0.07642	−0.06921 *	−0.07797 ***
	(−3.01)	(−1.27)	(−1.85)	(−2.98)
Good health	−0.18480 ***	−0.19826 ***	−0.02400	−0.12059 ***
	(−4.22)	(−2.7)	(−0.52)	(−3.75)
Monthly income Yes (ref)	-	-	-	-
Monthly income No	−0.04441	−0.00631	−0.04731	−0.03591
	(−1.44)	(−0.12)	(−1.47)	(−1.59)
Constant	−0.28197	0.54914	−0.47706	−0.22559
	(−0.28)	(0.31)	(−0.45)	(−0.3)
Number of obs	398	394	395	394
Log likelihood	−59.433033	−286.6758	−97.071186	56.594992
LR chi2(9)	85.31	94.14	31.77	123.14
Prob > chi2	0.0000	0.0000	0.0002	0.0000

NB: values in parentheses are t-values; *** = significance at 1%; ** = significance at 5%; * = significance at 10%.
